# TP53 Mutation Infers a Poor Prognosis and Is Correlated to Immunocytes Infiltration in Breast Cancer

**DOI:** 10.3389/fcell.2021.759154

**Published:** 2021-11-30

**Authors:** Ziwen Zhang, Ran Hao, Qiusheng Guo, Sheyu Zhang, Xiaojia Wang

**Affiliations:** ^1^ Department of Medical Oncology (Breast Cancer), Cancer Hospital of the University of Chinese Academy of Sciences, Zhejiang Cancer Hospital, Hangzhou, China; ^2^ Institute of Cancer and Basic Medicine, Chinese Academy of Sciences, Hangzhou, China; ^3^ School of Nursing, Hebei Medical University, Shijiazhuang, China; ^4^ The Second Clinical College of Zhejiang Chinese Medical University, Hangzhou, China; ^5^ School of Pharmaceutical Science and Technology, Tianjin University, Tianjin, China

**Keywords:** TP53, mutation, breast cancer, immunocytes infiltration, prognosis

## Abstract

**Background:** This study aimed to investigate the TP53 mutation, its potential immune features, its prognostic value, and its impact on immune infiltration in patients with breast cancer (BC).

**Methods:** We downloaded the somatic mutation data and clinicopathologic features of BC patients from the TCGA GDC database, UCSC Xena platform, and International Cancer Genome Consortium (ICGC) database. The association between the TP53 mutation, clinicopathology features, and overall survival (OS) in BC patients was analyzed. We evaluated the potential role of the TP53 mutation in the immune therapy response, including the tumor mutation burden (TMB), microsatellite instability (MSI), and tumor immune dysfunction and exclusion (TIDE). Moreover, ESTIMATE was employed to assess the ImmuneScore and StromalScore in BC patients. We also explored immunocyte infiltration related to the TP53 mutation and its potential mechanism. Immunohistochemistry (IHC) was performed to validate the association between the expression of CXCL1, CXCL10, and CCL20 and TP53 status.

**Results:** We found that the TP53 mutation was significantly associated with the shorter OS (*p* = 0.038) and was also an independent predictive factor of OS for BC patients (*p* < 0.001). Compared to that in the wild type group, the TP53-mutant group showed a higher TMB value (*P*< 0.001), MSI value (*p* = 0.077), and TIDE value (*p* < 0.001) with respect to BC patient immunotherapy. In addition, the ImmuneScore and StromalScore were both significantly increased in the TP53-mutant group (ImmuneScore: *p* < 0.001; StromalScore: *p* = 0.003). The results of CIBERSORT suggested that the TP53 mutation significantly promoted the infiltration of Tregs, T helper cells, and M0-type macrophages. KEGG and GSEA enrichment results suggested that the IL-17 signaling pathway and antigen processing and presentation pathways were significantly enriched in the TP53-mutant group. Importantly, based on IHC results of immune-related hub-genes, the chemokines CXCL1, CXCL10, and CCL20 were significantly upregulated in the TP53-mutant group in BC patients.

**Conclusion:** These results indicate that a TP53 mutation might serve as a biomarker for BC prognosis and is related to immunocyte infiltration in the tumor microenvironment.

## Introduction

Breast cancer (BC) is the most common malignancy in women and seriously threatens physical and mental health worldwide (Coughlin, 2019). It is currently estimated that there will be 276,480 newly diagnosed cases and 42,170 deaths from BC in the United States by 2020 (Le Blanc et al., 2020). According to histological characteristics, BC can be divided into HER2-positive, endocrine-dependent, and triple-negative breast cancer (TNBC) (Maughan et al., 2010). The treatment approaches should be based on the histological and molecular characteristics. Depending on the clinical subtype, therapeutic options include surgery, chemotherapy, endocrine therapy, and anti-HER2 targeting. However, 20–30% of BC cases still progress to distant metastases after diagnosis and treatment, and metastasis is the leading cause of death in approximately 90% of BC patients (Maughan et al., 2010; Britt et al., 2020). The tumor microenvironment (TME) is crucial for tumor progression and metastasis (Hinshaw and Shevde, 2019). The TME comprises not only cancer cells, but also the surrounding stromal cells and the tumor-infiltrating immune cells, and the immune cells play the leading role in the TME (Hinshaw and Shevde, 2019). With the development of immunotherapies with immune checkpoint blockade, the interaction between tumor and immune cells has come into focus (DeBerardinis, 2020). Recently, cancer treatment was revolutionized by immune checkpoint inhibitor (ICI) therapy owing to its durable clinical response, and ICI is usually considered in advanced metastatic BC (Santa-Maria and Nanda, 2018; Force et al., 2019). Nevertheless, some tumor tissues, especially TNBC, have a relatively low immune response after ICI treatment, which is mainly attributed to a “cold” immune microenvironment (Force et al., 2019). Thus, the exploration of new potential biomarkers to identify effective clinical therapy and improve the proportion of patients with BC responsive to ICI therapy must be solved.

The TP53 protein is a transcription factor that blocks tumor formation (Shahbandi et al., 2020). It is activated in response to several triggers, such as oncogene activation, DNA damage, hypoxia, and nutrient deprivation (Shahbandi et al., 2020). The TP53 protein serves as the guardian of the genome and monitors cell proliferation mainly by inducing DNA repair, cell-cycle arrest, and apoptosis (Baugh et al., 2018). Moreover, TP53 also contributes to other cellular processes, including angiogenesis, metabolism, stem cell maintenance, immune responses, and the cross talk between tumor cells and stromal cells TP53 (Baugh et al., 2018; Shahbandi et al., 2020). Nevertheless, the TP53 mutation is the most common mutation in BC, reported in 30% of BC and in 80% of TNBC cases (Silwal-Pandit et al., 2017). The TP53 mutation might alter the binding properties to its consensus sequence, and impair the transcriptional activation of TP53 target genes, which are involved in suppressing the tumor progression (Schon and Tischkowitz, 2018). Moreover, TP53-mutated tumors equip cells with novel tumor-promoting abilities, which include increased invasiveness, poor differentiation, and higher metastatic potential (Pitolli et al., 2019). Hancock et al. analyzed the molecular features of chemorefractory TNBC residual disease, and revealed that the TP53 mutations and MYC/TGFβ signaling pathway were the prominent drivers of recurrence, representing high-yield targets of the TP53 mutation (Hancock et al., 2019). These results suggest that TP53 mutation plays a vital prognostic role in BC.

Prior studies have indicated that TP53 status could shape the immune signatures by regulating the infiltration of the myeloid population, including neutrophils, macrophages, and monocytes (Blagih et al., 2020). Consequently, this upregulates the circulating neutrophils involved in tumor progression (Blagih et al., 2020). Further, cancer cells can modulate the TME through the secretion of cytokines and chemokines, and the TP53 mutation status drives the expression of CXCL1, CXCL10, and CCL20 (Addadi et al., 2010; Lowe et al., 2014). Other studies have suggested that significantly higher levels of immunocytes infiltrated into BC in patients with TP53 mutations compared to those with the wild-type phenotype, and TP53 mutation could promote the immunogenicity of tumors by regulating the TP53-related signaling pathways in BC (Li et al., 2019; Blagih et al., 2020). This might in part account for the mechanism through which TP53 mutations affect tumor immune infiltration. However, the significance of T53 mutations in BC therapy responses remains unclear. Presently, there is an urgent need to stratify patients according to TP53 status and evaluate the effects of TP53 mutations on predicting the efficacy of immunotherapy in BC.

In this study, we downloaded the somatic mutation data of BC from the TCGA GDC database and evaluated the relationship between the tumor mutation burden (TMB) and TP53 status in BC. Moreover, BC patients were divided into “TP53-mutant” and “TP53-wild-type” groups, to explore the differentially expressed genes (DEGs) related to TP53 mutations. Then, the functional enrichment analysis and gene-set enrichment analysis (GSEA) were performed to reveal the signaling pathways and biological processes associated with DEGs in TP53-mutant BC. We also constructed protein-protein interaction and mRNA-miRNA-lncRNA ceRNA network for hub-genes using Cytoscape and miRTarBase, respectively. Importantly, we also validated the association between hub-genes expression which related to TME and TP53 status by immunohistochemistry (IHC) in cancer tissues of BC patients. Further, we quantified the immune cells proportions in the TCGA-BRCA samples and compared the differences in the immune cell infiltration in tumor tissues between TP53-mutant and TP53-wild type (TP53-wt) groups. Additionally, we conducted Cox regression analysis to identify the prognostic role of the TP53 status with BC progression, and constructed a nomogram including TP53 status to predict the overall survival (OS) of BC patients.

## Materials and Methods

### Data Downloading and Bioinformatic Analyses

We obtained somatic mutation data of breast invasive carcinoma (BRCA) samples from the TCGA GDC database by choosing the “Masked Somatic Mutation” (https://portal.gdc.cancer.gov/) (Zhang et al., 2021). The preprocessing was employed with VarScan software and the somatic mutations were visualized using the MAFtools R package (Mayakonda et al., 2018). Then, we downloaded the RNA sequencing data (FPKM values) of the BC patients and subsequently converted FPKM values to TPM values. Moreover, the data were divided into the lncRNA and mRNA expression profiles. Further, we download the clinicopathologic features and outcomes in the same population from the UCSC Xena platform (http://xena.ucsc.edu/), such as sex, age, stage, and microsatellite instability (MSI) status (Speir et al., 2016). In addition, two datasets including somatic mutation and clinical data in BC patients were downloaded from the International Cancer Genome Consortium (ICGC) database (https://daco.icgc.org/), which were Breast Cancer-FR (BRCA-FR) and Breast Cancer-KR (BRCA-KR) (Zhang et al., 2019).

### Copy Number Alteration Analysis

To analyze the copy number variations (CNVs) of TP53 in TCGA-BRCA patients, we obtained the data of Masked Copy Number Segment using the TCGAbiolinks package in R language (Colaprico et al., 2016). The CNV data was processed using GISTIC 2.0 by performing the GenePattern5 function (Reich et al., 2006). During the analytical process, GISTIC 2.0 with default settings was used except for several parameter (i.e., the confidence was 0.99 and X chromosome was included before the analysis). Finally, the results of GISTIC 2.0 were visualized with the MAFtools R package.

### Correlations Between Somatic Mutation and Tumor Mutation Burden

To predict the response to ICI therapy caused by the TP53 mutation in BC patients, we computed the TMB, MSI, and tumor immune dysfunction and exclusion (TIDE, http://tide.dfci.harvard.edu) for each BC sample. The total number of the somatic mutations per megabase of the genome detected in the tumor was defined as the TMB (Yarchoan et al., 2017); the insertion or deletion of repeat units results in a change in the microsatellite length, which is referred to as MSI (Vilar and Gruber, 2010); TIDE is a computational framework that can evaluate the response to immunotherapy and predict tumor immune escape by analyzing the gene expression profiles of cancer cases (Jiang et al., 2018). We calculated all TMB, MSI, and TIDE values for each sample, and compared their differences between patients with wild-type TP53 and those with mutant TP53 using a Wilcoxon rank-sum test.

### Relationship Between Clinical Features and Differentially Expressed Genes

To explore the significance of mutant TP53 in BC progression, we classified the TCGA patients into “TP53-mutant” and “TP53-wt” groups. The holistic analysis was employed by principal component analysis (PCA), which is a multivariate statistical technique under the broad title of factor analysis, that focus on pattern recognition and signal processing (Ringnér, 2008). PCA was conducted with the R packages factoextra and FactoMineR. DEGs were determined using the Bioconductor R package DESeq2 (Love et al., 2014), and the threshold for DEGs was *p* < 0.01 and |logFC| > 1.5. The results were presented in heatmap and volcano plots.

### Functional Enrichment Analysis and Gene-Set Enrichment Analysis

Gene ontology (GO) analysis is a common bioinformatics tool applied in large-scale functional enrichment studies that can annotate genes and analyze the biological process, cellular component, and molecular function of these genes (Yu et al., 2012). Kyoto Encyclopedia of Genes and Genomes (KEGG, http://www.genome.jp/kegg/) is a database to explore the comprehensive biological systems and functions generated by experimental techniques in high-throughput biology from massive molecular datasets (Yu et al., 2012). The GO annotation and KEGG pathway enrichment analyses of signature genes was implemented using the ClusterProfiler package and the DAVID online database (Yu et al., 2012). Results with a false discovery rate (FDR) less than 0.05 were considered statistically significant.

To investigate the differences in biological processes between TP53-mutant and TP53-wt groups, we performed GSEA, based on the gene expression profile of the TCGA-BRCA dataset. GSEA is also a functional enrichment analysis, based on a predefined set of genes between two groups, which can determine whether there is a statistical difference (Subramanian et al., 2005). It is used frequently in analyzing the enrichment of signaling pathways and biological processes. The geneset of c2. cp.kegg.v6.2.-symbols was downloaded from the Molecular Signature Database (MsigDB, http://software.broadinstitute.org/gsea/msigdb/). GSEA was performed, and adjusted *p*-values less than 0.05 were regarded as statistically significant.

### Comparison of Immune Cell Infiltration and Immune Scores Between Two Groups

To quantify the immune cell proportions in the TCGA-BRCA samples, we used the CIBERSORT algorithm (https://cibersort.stanford.edu/) and the LM22 gene signature matrix (Newman et al., 2015). Highly sensitive and specific discrimination was performed for the phenotypes of 22 immunocytes (T cells, B cells, natural killer cells, and macrophages) in the TME (Hinshaw and Shevde, 2019). CIBERSORT was run to deconvolute samples, and used the expression values of a set of reference genes (547 genes), which were considered the minimal representative values for each type of cells. Based on these values, we deduced the cell type proportioning from the data of samples with mixed cells. Thus, we analyzed the effect of TP53 gene mutations on immune cell infiltration in TCGA-BRCA patients.

Meanwhile, the ESTIMATE algorithm was applied to assess the immune infiltration levels of BC patients according to the interpretation of gene expression profiles (Yoshihara et al., 2013). The ImmuneScore and StromalScore were calculated for each sample using the using the ESTIMATE package in R (https://www.r-project.org/). We performed Mann-Whitney U tests to compare the differences in immune cell infiltration in tumor tissues between TP53-mutant and TP53-wt groups.

### Construction of Protein-Protein Interaction Network and Identification of Hub-Genes

In this study, we implemented the STRING (https://string-db.org) (Szklarczyk et al., 2019) to infer the protein-protein interaction (PPI) network. STRING is an online tool that can predict protein-protein interactions and construct the PPI network of selected genes. Interactions with a confidence score greater than 0.7 were included to construct the PPI network in Cytoscape software (Version 3.7.2). We defined the high-density areas as hub-genes based on the vertex-weighting scheme by using the MCODE plugin (Shannon et al., 2003).

### Construction of mRNA-miRNA-lncRNA ceRNA Network

The miRNA-mRNA interaction data was downloaded from the mirTarBase (http://mirtarbase.mbc.nctu.edu.tw/index.php) (Hsu et al., 2011). Then, we predicted the target miRNAs of the hub-genes from the PPI network, and carried out using the miRTarBase (http://mirtarbase.mbc.nctu.edu.tw) (Hsu et al., 2011). Moreover, the regulatory relationships between miRNA and lncRNA were further established. Based on these hub gene-miRNA pairs and miRNA-lncRNA pairs, a ceRNA network for mRNA-miRNA-lncRNA was illustrated using Cytoscape software (version 3.7.2) (Shannon et al., 2003).

### Immunohistochemistry

To validate the association between the expression of CXCL1, CXCL10, and CCL20 and TP53 status, we collected 10 cancer tissues with TP53 mutation and 10 tissues without mutation from BC patients. We performed IHC to compare the level of CXCL1, CXCL10, and CCL20 between two groups. IHC was performed as previously described (Wang et al., 2021), with antibodies specific for TP53 (Affinity, 1:100), CXCL1 (Affinity, 1:100), CXCL10 (Affinity, 1:100), or CCL20 (Affinity, 1:100). Pictures were taken with a microscope (Nikon DS-Ri2, Tokyo, Japan). Pathological samples were evaluated and scored separately by two qualified pathologists. The IHC scoring is as follows: 0 for no staining, 1+, 2+, 3+, and 4 + for 1–24, 25–49%, 50–74%, and over 75% staining intensity, respectively.

### Analysis of Anti-Cancer Drugs Sensitivity

Genomics of Drugs Sensitivity in Cancer (GDSC) is a public online database (http://www.cancerrxgene.org/downloads/) and is used to determine anticancer drug response and somatic mutations in cancer (Yang et al., 2013). We identified the association between TP53 mutations and anticancer drug sensitivity in BC patients, based on the data of the gene mutation status in cancer cell lines and IC50 values of anticancer drugs.

### Construction of TP53-Mutation Prognostic Model

To identify the prognostic role of TP53 status based on clinicopathological features, we analyzed the OS rate by conducting univariate and multivariate Cox regression analyses to test the risk score. The potential prognostic parameters were included to construct a nomogram using the TCGA-BRCA datasets. We constructed the nomogram using the rms R package. To analyze the performance of models, a calibration plot was graphically mapped by the nomogram predicted vs. observed probability. Moreover, the concordance index (C-index) was commonly obtained to quantitatively examine the discrimination ability of the nomogram.

### Statistical Analysis

In this study, all data processing and analysis were carried out using R software (Version 4.0.2). For continuous variables, a Student’s t-test was used to compare the means between the normally distributed variables, whereas a Mann-Whitney test was used for the variables that were not normally distributed. Moreover, a Chi-square test or Fisher exact test was used for discontinuous variables. The correlations among genes were determined by Pearson correlation analysis. Prognostic analysis was performed using the R package survival. The Kaplan-Meier curves were plotted to show the survival time of BC patients, and the log-rank test was used for the survival comparisons between the two groups. The independent prognostic factors in BC were identified using univariate Cox regression and multivariate Cox regression analyses. We plotted the receiver operating characteristic (ROC) curves using the pROC R package (Robin et al., 2011). The area under the ROC curve (AUC) was calculated to assess the prognostic risk scores (Robin et al., 2011). A two-sided *p* value less than 0.05 was considered as statistically significant.

## Results

### Overall Mutation Analyses of Breast Cancer Patients

**FIGURE 1:**
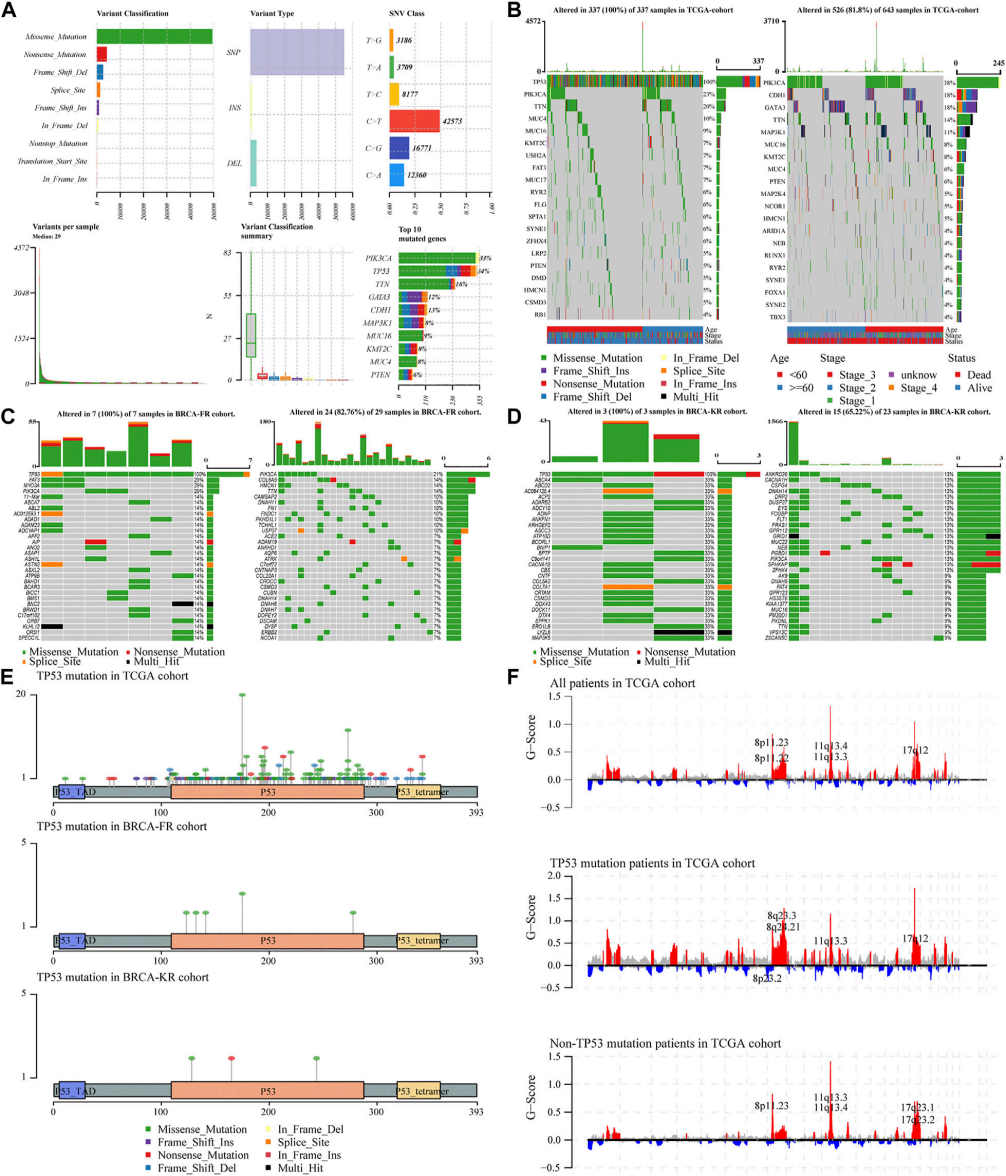
Characteristic of TP53 mutation in TCGA-BRAC patients. Overall information of somatic mutation of BRCA patients **(A)**; the top 30 significant mutations were found in the TCGA-BRCA cohort, of which the left side was TP53-mutant group and the right side was TP53-wt group **(B)**; the distribution of the top 30 significant mutations in the dataset of BRCA-FR **(C)** and BRCA- KR **(D)**; the distribution of amino acids in TP53 protein in TCGA-BRCA, BRCA-FR, and BRCA-KR data sets **(E)**; the results of CNV for TCGA-BRCA were visualized by MAFtools **(F)**. SNP, single nucleotide polymorphism; SNV, single nucleotide variant; CNV, copy number variation.

To analyze the effects of TP53 mutations on the genomic mutations in BC patients, we downloaded three BRCA datasets from TCGA and ICGC databases (n = 943). First, we evaluated the mutation profile in BRCA patients as shown in Figure 1A. The results indicated that the missense mutations accounted for a major portion, single nucleotide polymorphisms (SNPs) were more often observed than insertion-deletion (indel) mutations, and the C > T single nucleotide variants were the most common variant in BC patients. The frequency of TP53 mutations was the second most in all the BRCA patients. Subsequently, we subdivided all patients into two groups, TP53-mutant and TP53-wt groups, according to the TP53 status. The somatic mutations of BRCA samples were calculated and visualized by the “Maftools” R package, and were presented in Figure 1B. The waterfall plots presented the mutation profile of associated genes (Figure 1B for TCGA-BRCA; Figure 1C for BRCA-FR; and Figure 1D for BRCA-KR). Moreover, the amino acid substitutions in the TP53 gene were evaluated and shown in Figure 1E. The location of each amino acid variant was corresponding to the coordinate axis below. The mutation type was distinguished by different colors, and the tag indicates the meaning of each color. The results showed that the main mutant form of TP53 amino acid was missense mutation in all three datasets. We also separated TCGA-BRCA patients into TP53-mutant and TP53-wt groupsTP53, and analyzed the CNV status. The data were analyzed via GISTIC 2.0 to obtain gene-level estimates of CNV, with the default settings except for several parameters (e.g., confidence: 0.99; X chromosome was not excluded from the analysis). Finally, the GISTIC 2.0 output was visualized using the MAFtools package, and shown in Figure 1F. This indicated that significant alterations in CNV levels located in related genes were observed in the TP53-mutant group.

### Association Between TP53 Mutation and Immunotherapy Indicators

**FIGURE 2:**
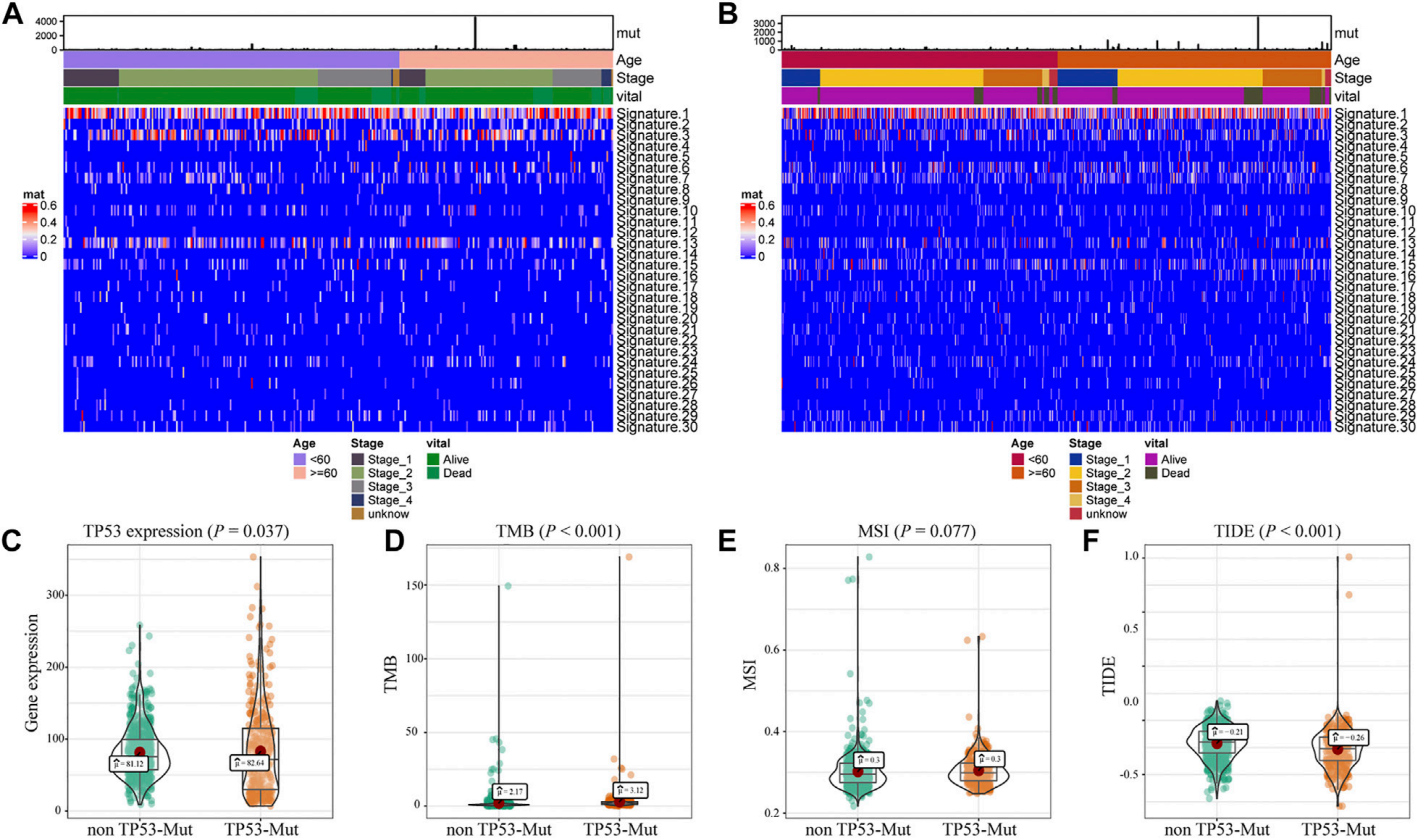
Analysis of biological characteristics of TP53 mutation in BC patients. The cluster analysis of cosmic signature in patients with TP53 mutation and the clinical features of patients were shown by the heatmap **(A)**; the cluster analysis of cosmic signature in patients without TP53 mutation in TCGA-BRCA dataset **(B)**; compared with TP53-wt group, the expression level of TP53 was significantly increased in TP53 mutation group of the BC patients **(C)**; the TMB level of TP53-mutation patients was significantly increased **(D)**; the MSI value of TP53 mutation patients was significantly increased **(E)**; TIDE in patients with TP53 mutation was significantly higher **(F)**. TMB, tumor mutation burden; MSI, microsatellite instability; TIDE, tumor immune dysfunction and exclusion.

Further, we explored the biological effect of TP53 mutations based on the mutational signature analysis. According to the biological characteristics, somatic mutational processes could be characterized by the mutation patterns, and 96 mutation patterns were translated into 30 different mutational signatures (Alexandrov et al., 2020). The results indicated that significant changes in Signature 1, 3, and 13 were observed compared to those in the TP53-wt group (Figures 2A,B). In addition, compared with that in the TP53-wt group, the level of the TP53 gene was substantially increased in the TP53-mutant group (*p* = 0.037; Figure 2C), and the TMB value (*p* < 0.001; Figure 2D), MSI value (*p* = 0.077; Figure 2D), and TIDE score for immunotherapy (*p* < 0.001; Figure 2E) were also elevated in the TP53-mutant group.

### Analysis of Drug Sensitivity in Breast Cancer Patients with the TP53 Mutation

**FIGURE 3:**
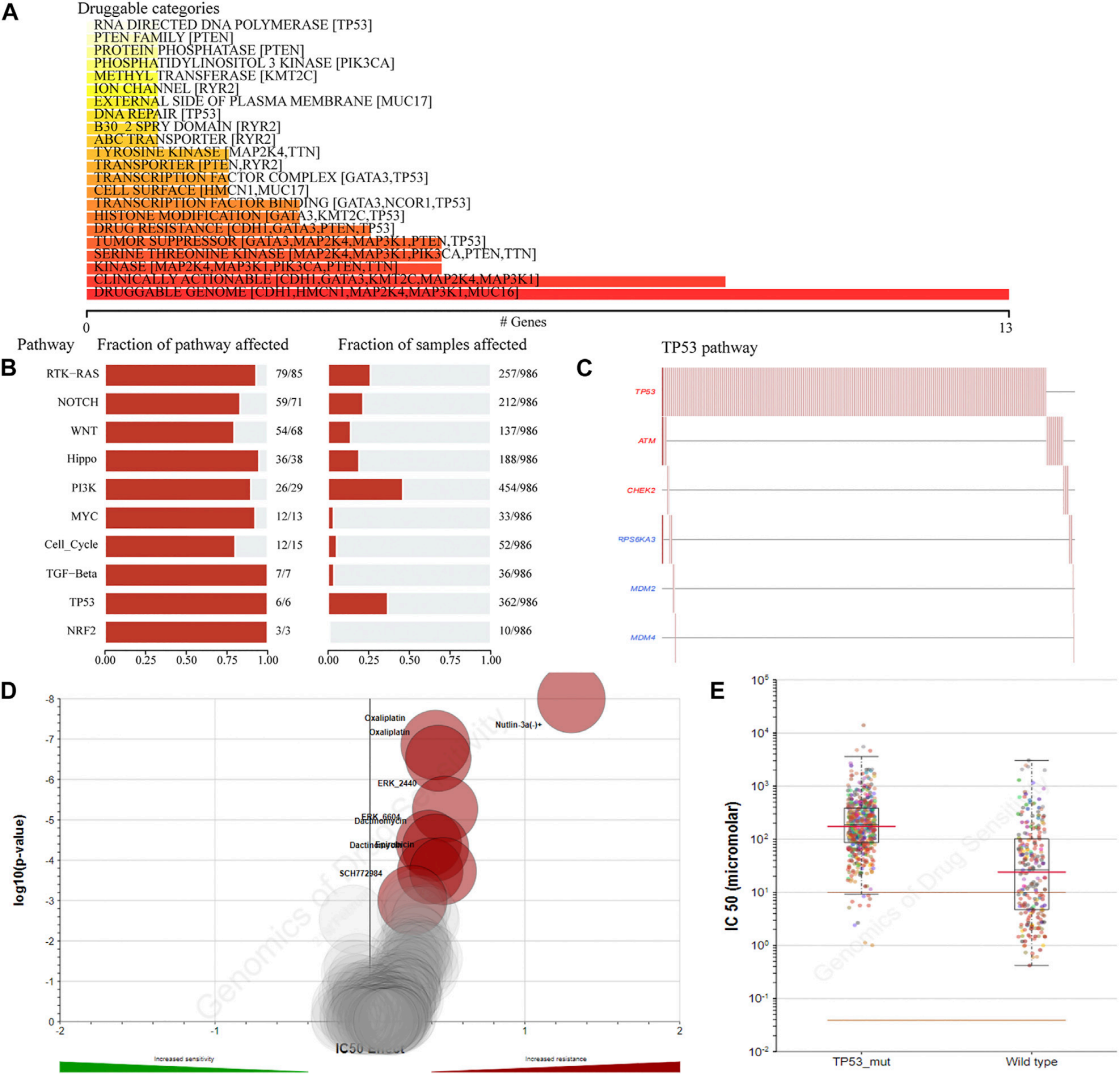
The drug sensitivity caused by TP53 mutation. The relationship between gene mutation level and different kinds of drugs in TCGA-BRCA dataset was analyzed **(A)**; analysis of gene mutation level in different carcinogenic signaling pathways in TCGA-BRCA dataset **(B)**; mutation distribution of six major genes in TP53 signaling pathway in TCGA-BRCA patients **(C)**; the volcano map shows the sensitivity analysis of TP53 gene mutation to different anticancer chemotherapy drugs; the red circle indicates that TP53 gene mutation leads to the decrease of drug sensitivity **(D)**; compared with TP53-wt group, the IC50 value of nutlin-3a (-) in patients with TP53 mutation was significantly higher **(E)**. wt, wild type.

To detect the effect of TP53 mutations on drug sensitivity in BC patients, we assessed the correlation between TP53 mutations and IC50 values of molecules from the GDSC database. The result showed that multiple drugs related to the frequency of TP53 mutation (Figure 3A). The pathway analysis revealed that the TP53 pathway was significantly enriched (Figure 3B), and the high mutation rates of 6 genes in this pathway were also prevalent in BC patients (Figure 3C). Moreover, the TP53 mutation had some effect on BC sensitivity to multiple chemotherapy agents and small molecule substances (Figure 3D), especially to Nutlin-3a (Figure 3E).

### Differential Gene Expression Analysis in Breast Cancer Patients

**FIGURE 4:**
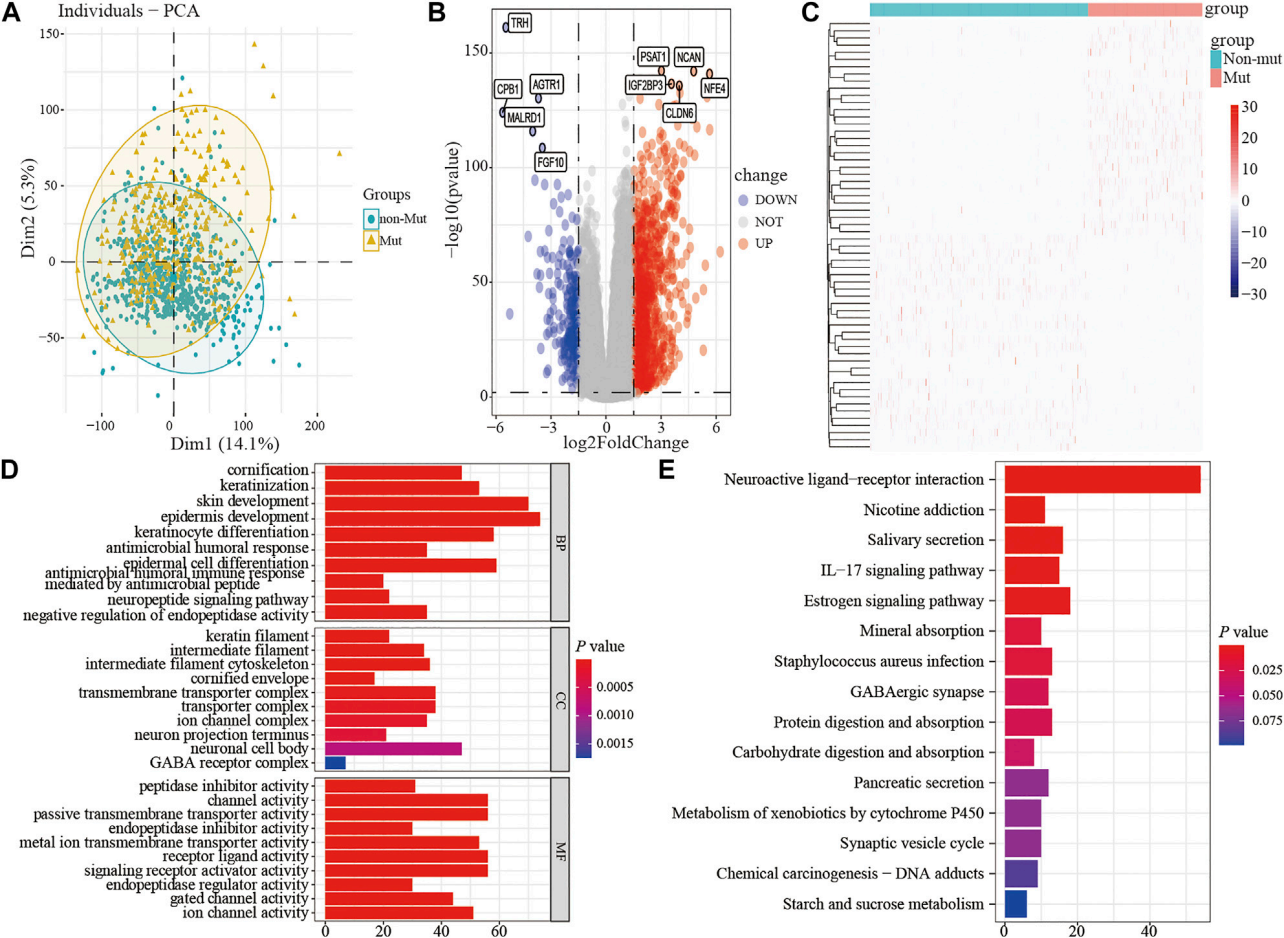
The differential expression and functional enrichment analysis based on TP53 mutation. PCA was performed between the patients with mutation and non-mutation **(A)**; volcano map and heatmap showed the expression of DEGs between TP53-mutation and TP53-wt group **(B-C)**; the results of GO analysis included the cell component **(D)**, biological process **(E)**, and molecular function **(F)** terms; the results of KEGG analysis that differentially expressed genes were involved in biological related signaling pathways **(G)**. PCA, principal component analysis; wt, wild type.

To assess the effect of the TP53 mutation on BC tumorigenesis, the TCAG-BRCA patients were separated into TP53-mutant and TP53-wt groups. As shown in Table 1, TP53 mutation status was significantly correlated with a younger age (<60 vs. ≥ 60, *p* = 0.007) and earlier M stage (M0 vs. MX, *p* = 0.007). As evaluated by PCA analysis, significant differences were shown (*p* < 0.05) between TP53-mutant and TP53-wt groups (Figure 4A). Moreover, DEGs analysis identified that 845 upregulated DEGs and 237 downregulated DEGs were associated with the TP53 mutation (|log2 fold change|> 1.5 and (adjust) *p*-value < 0.01; Figures 4B,C).

**TABLE 1:** Association between TP53 status and clinical pathologic features in TCGA-BRCA patients.

Variables	All patients (n = 943)	TP53-wt (n = 624)	TP53-mutant (n = 319)	*p* value
Age	—	—	—	0.007
＜60	517 (54.8%)	322 (51.6%)	195 (61.1%)	—
≥60	426 (45.2%)	302 (48.4%)	124 (38.9%)	—
Pathologic stage	—	—	—	0.961
I and II	713 (75.6%)	471 (75.5%)	242 (75.9%)	—
III and IV and X	230 (24.4%)	153 (24.5%)	77 (24.1%)	—
T	—	—	—	0.719
T1 and T2	803 (85.2%)	529 (84.8%)	274 (85.9%)	—
T3 and T4 and TX	140 (14.8%)	95 (15.2%)	45 (14.1%)	—
N	—	—	—	0.601
N0 and N1	767 (81.3%)	511 (81.9%)	256 (80.3%)	—
N2 and N3 and NX	176 (18.7%)	113 (18.1%)	63 (19.7%)	—
M	—	—	—	0.042
M0	788 (83.6%)	510 (81.7%)	278 (87.1%)	—
M1 and MX	155 (16.4%)	114 (18.3%)	41 (12.9%)	—

Subsequently, to analyze the cellular functions of 1082 DEGs, we conducted GO and KEGG enrichment analyses using the R package clusterProfiler. The results of the GO analysis demonstrated that DEGs were involved in the biological processes of cornification, keratinization, skin development, intermediate filament cytoskeleton, and peptidase inhibitor activity (Table 2; Figures 4D–F). KEGG pathway analysis suggested that the immune-related DEGs were significantly enriched in neuroactive ligand-receptor interaction, nicotine addiction, salivary secretion, and the IL-17 signaling pathway (Table 3; Figure 4G). Besides, the GSEA results of the TP53-mutant group revealed that the significant pathways (*p* < 0.05 and FDR q-value < 0.25) were enriched in focal adhesion, ribosome, antigen processing and presentation, and ECM receptor interaction, and the details are shown in Table 4 and Supplementary Figure S1A, S1B.

**TABLE 2:** Top three clusters with their representative enriched terms of GO analysis.

GO	ID	Description	Count	*p* value	Gene
BP	GO:0070268	Cornification	47	4.11E-32	KRT16/KRT83/PI3/DSG1/DSC2/KRT9/KRT6B/KRT79/TGM1/KRT86/KRT6A/KLK5/DSG3/KRT6C/CASP14/KLK14/SPINK5/DSC3/KRT81/PKP1/TGM5/KRT37/KRT4/SPINK6/KRT34/KRT23/KRT78/KRT5/KRT31/KRT1/KRT3/KRT17/IVL/KRT14/KRT75/KRT35/KRT84/LIPK/KRT85/KRT77/KRT82/SPRR1B/KRT33B/SPRR2G/SPRR2D/SPRR2E/LCE3D
BP	GO:0031424	Keratinization	53	3.39E-22	KRT16/KRT83/PI3/DSG1/DSC2/KRT9/KRT6B/KRT79/TGM1/KRT86/CDH3/KRT6A/KLK5/DSG3/KRT6C/CASP14/KLK14/SPINK5/DSC3/KRT81/PKP1/TGM5/KRT37/KRT4/SPINK6/KRT34/KRT23/KRT78/KRT5/KRT31/KRT1/KRT3/KRT17/IVL/KRT14/KRT75/KRT35/KRT84/KRTAP3-3/LIPK/KRT85/KRT77/KRT82/KRTAP1-1/LCE3A/SPRR1B/KRT33B/SPRR2G/SPRR2D/SPRR2E/LCE3D/SPRR4/KRTAP4-1
BP	GO:0043588	Skin development	70	1.12E-19	FOXC1/FGF10/KRT16/KRT83/PI3/DSG1/DSC2/EGFR/CTSV/KRT9/KRT6B/GAL/KRT79/TGM1/KRT86/CDH3/KRT6A/KLK5/FERMT1/CLDN1/SCEL/GJB3/DSG3/KRT6C/FOXQ1/CASP14/LHX2/KLK14/SOSTDC1/SPINK5/LGR5/DSC3/EDAR/KRT81/DKK1/PKP1/TGM5/KRT37/KRT4/SPINK6/KRT34/KRT23/KRT78/KRT5/KRT31/KRT1/KRT3/KRT17/IVL/KRT14/KRT75/KRT35/KRT84/KRTAP3-3/LIPK/KRT85/KRT77/KRT82/S100A7/KRTAP1-1/LCE3A/SPRR1B/KRT33B/SPRR2G/SPRR2D/SERPINB13/SPRR2E/LCE3D/SPRR4/KRTAP4-1
CC	GO:0045095	Keratin filament	22	8.00E-10	KRT83/KRT6B/KRT79/KRT86/KRT6A/KRT6C/CASP14/KRT81/KRT4/KRT78/KRT5/KRT1/KRT3/KRT14/KRT75/KRT84/KRTAP3-3/KRT85/KRT77/KRT82/KRTAP1-1/KRTAP4-1
CC	GO:0005882	intermediate filament	34	1.23E-09	INA/KRT16/KRT83/KRT9/KRT6B/KRT79/KRT86/KRT6A/KRT6C/KRT222/CASP14/KRT81/PKP1/KRT37/KRT4/KRT34/KRT23/KRT78/KRT5/KRT31/KRT1/KRT3/KRT17/KRT14/KRT75/KRT35/KRT84/KRTAP3-3/KRT85/KRT77/KRT82/KRTAP1-1/KRT33B/KRTAP4-1
CC	GO:0045111	Intermediate filament cytoskeleton	36	6.84E-09	INA/KRT16/KRT83/KRT9/KRT6B/KRT79/SLC1A6/KRT86/KRT6A/S100A8/KRT6C/KRT222/CASP14/KRT81/PKP1/KRT37/KRT4/KRT34/KRT23/KRT78/KRT5/KRT31/KRT1/KRT3/KRT17/KRT14/KRT75/KRT35/KRT84/KRTAP3-3/KRT85/KRT77/KRT82/KRTAP1-1/KRT33B/KRTAP4-1
MF	GO:0030414	Peptidase inhibitor activity	31	3.30E-09	A2ML1/RARRES1/PI3/SLPI/NLRP7/CST9L/SERPINB7/CST5/SERPINB5/SPINK5/CST2/CARD17/UMODL1/HMSD/CST9/SPINK6/SERPINB2/SERPINB4/SERPINA11/SERPINB12/MT3/CST4/CARD18/FETUB/SMR3B/SERPINA6/SERPINB3/OPRPN/SMR3A/SERPINB13/CSN2
MF	GO:0015267	Channel activity	56	4.50E-09	SLC26A9/TRPM8/TTYH1/TMC3/GRIA1/KCNS1/KCNQ4/KCNK5/KCNG1/CLCN4/KCNB2/CHRNA9/GABRP/GRIA2/KCNE4/GABRA5/CHRNA5/HTR3A/GABRE/CNGB1/GJB3/KCNE5/KCNK9/CNGA1/GRIN2B/CACNA1B/TRPV3/GLRA3/SCN7A/TRPV6/CNGA3/KCNH1/GJB7/KCNC1/AQP5/ABCC8/KCNJ4/CLIC6/KCNC2/GABRG3/GABRQ/KCNV1/KCNF1/UNC80/GJB4/CLCA2/ASIC2/OTOP1/KCNJ3/CACNG5/GABRA3/KCNJ18/KCNK16/AQP12B/HTR3B/CLCA1
MF	GO:0022803	Passive transmembrane transporter activity	56	4.86E-09	SLC26A9/TRPM8/TTYH1/TMC3/GRIA1/KCNS1/KCNQ4/KCNK5/KCNG1/CLCN4/KCNB2/CHRNA9/GABRP/GRIA2/KCNE4/GABRA5/CHRNA5/HTR3A/GABRE/CNGB1/GJB3/KCNE5/KCNK9/CNGA1/GRIN2B/CACNA1B/TRPV3/GLRA3/SCN7A/TRPV6/CNGA3/KCNH1/GJB7/KCNC1/AQP5/ABCC8/KCNJ4/CLIC6/KCNC2/GABRG3/GABRQ/KCNV1/KCNF1/UNC80/GJB4/CLCA2/ASIC2/OTOP1/KCNJ3/CACNG5/GABRA3/KCNJ18/KCNK16/AQP12B/HTR3B/CLCA1

**TABLE 3:** Top nine clusters with their representative enriched terms of KEGG analysis.

ID	Description	Count	*p* value	Gene
hsa04080	Neuroactive ligand-receptor interaction	53	1.35E-15	7,200/185/887/165,829/1131/9,568/2890/4,986/51,083/6019/55,584/2568/2,891/4886/2,558/1138/5,697/2564/1394/64,106/5746/5,646/3362/2,904/8001/4,923/2692/4,887/10,874/7434/2,567/55,879/2691/4,889/553/6863/797/4922/1081/57,152/885/5540/4,543/117,579/2556/7,201/5173/796/84,539/9248/3,358/1443/2,689	
hsa05033	Nicotine addiction	11	1.12E-06	2,890/2568/2,891/2558/2,564/2904/774/2567/55,879/2556/57,084	
hsa04970	Salivary secretion	16	4.07E-06	1131/4025/1473/477/1470/492/55,503/480/362/653,247/1755/3346/1472/51,806/277/5,542	
hsa04657	IL-17 signaling pathway	15	2.09E-05	6,374/2919/6,279/3627/6,364/3934/6,354/6280/3,576/5596/4,312/6372/338,324/6278/1673	
hsa04915	Estrogen signaling pathway	18	5.38E-05	3,868/9568/1956/3857/399,694/2099/8688/3,885/25,984/7031/5,241/3881/3,872/3861/3,886/3760/51,806/3,884	
hsa05150	*Staphylococcus aureus* infection	13	0.000403	3,868/1828/3857/1672/8688/3,885/25,984/3881/3,872/3861/3,886/3884/1673	
hsa04727	GABAergic synapse	12	0.000695	9,568/2568/18/2558/2,564/774/10,991/2571/2,567/6538/55,879/2,556	
hsa04974	Protein digestion and absorption	13	0.000799	1302/1360/1299/59,272/6564/1297/8645/169,044/136,227/5646/477/480/256,076	
hsa04973	Carbohydrate digestion and absorption	8	0.001174	93,432/3938/8,972/477/480/80,201/57,818/277	

**TABLE 4:** KEGG pathways enriched in TP53-mutant and TP53-wt groups by using GSEA analysis.

Name	Size	Enrichment Score	NES	*p* value	Leading edge
KEGG_RIBOSOME	87	0.946565	1.633661	1.00E-10	tags = 84%, list = 4%, signal = 81%
KEGG_FOCAL_ADHESION	199	0.835	1.468514	2.31E-09	tags = 32%, list = 7%, signal = 30%
KEGG_ECM_RECEPTOR_INTERACTION	83	0.88544	1.526534	1.46E-06	tags = 35%, list = 5%, signal = 33%
KEGG_ANTIGEN_PROCESSING_AND_PRESENTATION	80	0.869832	1.498648	7.79E-06	tags = 38%, list = 9%, signal = 34%
KEGG_REGULATION_OF_ACTIN_CYTOSKELETON	212	0.767001	1.350277	3.37E-05	tags = 37%, list = 14%, signal = 32%
KEGG_ARRHYTHMOGENIC_RIGHT_VENTRICULAR_CARDIOMYOPATHY_ARVC	74	0.852098	1.467021	0.000171	tags = 22%, list = 5%, signal = 21%
KEGG_VIRAL_MYOCARDITIS	68	0.861515	1.480648	0.000171	tags = 24%, list = 4%, signal = 23%
KEGG_PATHOGENIC_ESCHERICHIA_COLI_INFECTION	55	0.878604	1.504304	0.000189	tags = 36%, list = 4%, signal = 35%
KEGG_LEUKOCYTE_TRANSENDOTHELIAL_MIGRATION	115	0.799328	1.39047	0.000193	tags = 32%, list = 10%, signal = 29%
KEGG_DILATED_CARDIOMYOPATHY	90	0.81603	1.409257	0.000393	tags = 13%, list = 5%, signal = 13%

### Protein-Protein Interaction and ceRNA Network

**FIGURE 5:**
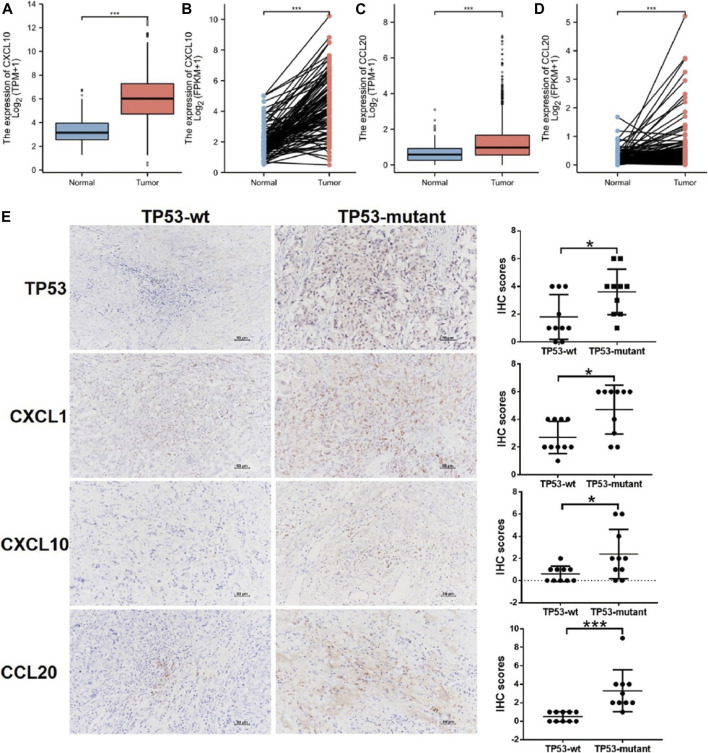
Differential expression of CXCL1, CXCL10, CCL20, and TP53. **(A)** CXCL10 expression in BC tissues (n = 1109) vs. the normal tissues (n = 113). **(B)** CXCL10 expression in breast cancer tissues (n = 112) vs. the paired-paracancerous tissues (n = 112). **(C)** CCL20 expression in BC tissues (n = 1109) vs. the normal tissues (n = 113). **(D)** CCL20 expression in BC tissues (n = 112) vs. the paired-paracancerous tissues (n = 112). **(E)** IHC analysis of the expression of TP53, CXCL1, CXCL10, and CCL20 between TP53-wt (n = 10) and TP53-mutant (n = 10) group in BC tissues. BC, breast cancer.

The PPI network of DEGs was constructed using the STRING online database (Supplementary Figure S2A), and the results were imported into Cytoscape software for further analysis (Supplementary Figure S2B); the red color represented up-regulated gene expression and the blue color represented down-regulated gene expression. Then, we used the plugin MCODE in Cytoscape to analyze the important modules. In the regions of high density, the central nodes were identified as hub-genes (Supplementary Figure S2C). Based on the information of miRNA-mRNA interactions in the miRTarBase, we predicted the miRNAs associated with the hub-genes, and lncRNA associated with the miRNAs. Thus, the mRNA-miRNA-lncRNA ceRNA network was constructed based on the predicted relationship shown in Supplementary Figure S2D. The results above indicated that the chemokines CXCL1, CXCL10, and CCL20 was significantly upregulated in the TP53-mutant group (Supplementary Figure S2C, S2D). Further, the CXCL10 and CCL20 expression level was lower in BC tissues (TCGA-BRCA patients) compared with normal tissues (Figures 5A,B). We also examined the expression level of them in paired tissue samples. The results indicated that the level of CXCL10 and CCL20 in BC tissues was also significantly lower than those in paired samples (Figures 5C,D).

### Relationship Between Hub-Genes Expression and TP53 Status

We determined the effects of TP53 mutation on the expression of hub-genes by IHC in BC tissues, including CXCL1, CXCL10, and CCL20. As shown in Figure 5E, the upregulated expression of CXCL1, was significantly associated with TP53 mutation (*p* < 0.05). The similar results were also found for the expression of CXCL10, CCL20, and TP53 (*p* < 0.05; Figure 5E).

### Association Between TP53 Mutation and Breast Cancer Immunogenicity

**FIGURE 6:**
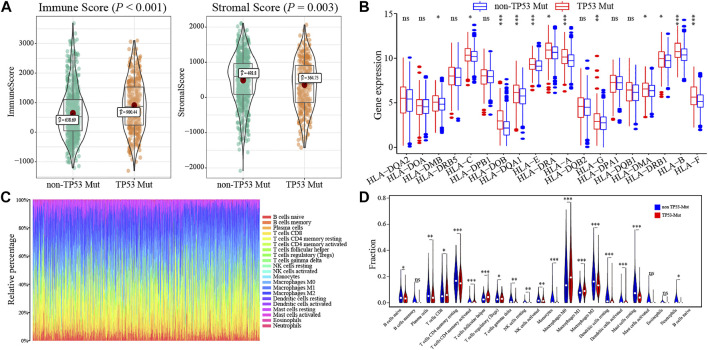
The association between TP53 mutation and immunological phenotype in TCGA-BRCA patients. In TCGA-BRCA dataset, the ImmuneScore and StromalScore of TP53-mutant patients were significantly increased **(A)**; there was significant difference in the expression of HLA family genes between the two groups **(B)**; the histogram showed the proportion of 22 different immune cells in TCGA-BRCA patients **(C)**; the results of the difference analysis showed that the infiltration level of various immune cells was significantly different in TP53-mutant and TP53-wt group **(D)**.

To determine how the TP53 mutation influences BC immunogenicity, we compared the expression differences in immune-related genes and stromal-related genes between the TP53-mutant group and TP53-wt group. The results indicated that in the mutation group, the levels of the ImmuneScore and StromalScore were both significantly increased (ImmuneScore: *p* < 0.001; StromalScore: *p* = 0.003; Figure 6A). Moreover, the expression of multiple HLA gene families was significantly upregulated in the mutation group (Figure 6B). Next, we used CIBERSORT to evaluate the composition ratio of 22 immune cell types in each BC sample and the result showed individual differences (Figure 6C). We also compared the levels of 22 immune cells between the TP53-mutant group and TP53-wt group. The results demonstrated that the proportions of Tregs, T helper cells, and M0 type macrophages were significantly upregulated in the TP53-mutant group (Figure 6D, *p* < 0.05), whereas the proportion of resting CD4^+^ T cell and M2-type macrophages was lower (Figure 6D, *p* < 0.05).

### Association Between the TP53 Status and Clinical Outcomes

**FIGURE 7:**
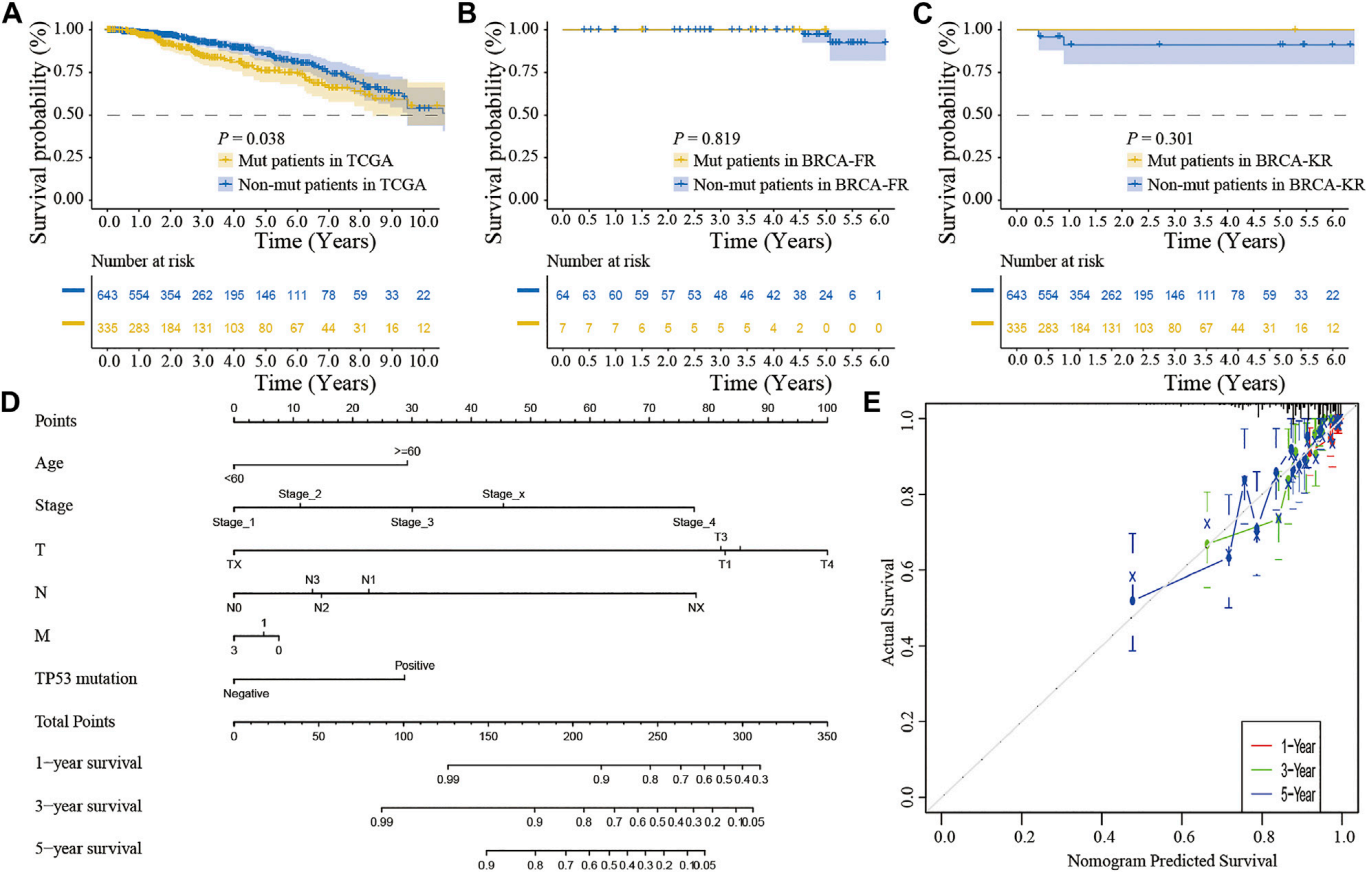
The clinical significance of TP53 mutation in breast cancer patients. The survival analysis showed that the prognosis of patients with TP53 mutation was better in TCGA-BRCA dataset **(A)**; in BRCA-FR and BRCA-KR datasets, there was no significant correlation between TP53 mutation and disease prognosis **(B-C)**; the mutation of TP53 gene was combined with clinicopathological characteristics to construct the nomogram **(D)**; the calibration curve of the TP53 mutation was to evaluate the discrimination ability of the nomogram **(E)**. The horizontal coordinate was the survival predicted by the nomogram, and the vertical coordinate was the actual observed survival.

We also performed the Kaplan-Meier analysis to assess the prognostic significance of TSPOAP1-AS1 expression. In the TCGA-BRCA patients, the TP53 mutation was significantly associated with a shorter OS (*p* = 0.038; Figure 7A), whereas there was no significance for BRCA-FR (*p* = 0.819; Figure 7B) and BRCA-KR (*p* = 0.301; Figure 7C) patients. Then, to further confirm the prognostic value of the TP53 mutation, we conducted univariate and multivariate Cox regression analyses for OS. The results revealed that TP53 mutation (*p* = 0.0298), age (*p* < 0.001), tumor stage (*p* < 0.001), T stage (*p* = 0.01), N stage (*p* < 0.001), and M stage (*p* < 0.001) were correlated with BC prognosis (Table 5). Then, these variables were included to build the multivariable Cox models of OS (Table 5). The TP53 mutation remained independently associated with OS [HR: 1.76 (1.24–2.50), *p* = 0.002], which was also true for age [HR: 1.94 (1.37–2.76), *p* < 0.001], tumor stage [HR: 2.46 (1.26–4.80), *p* = 0.009], and M stage [HR: 1.67 (1.05–2.66), *p* = 0.03]. These results revealed that the TP53 mutation is an independent predictive factor of OS in BC patients. Further, to develop a clinical quantitative tool to predict the OS for BC patients, a nomogram was constructed based on the results of multivariable cox regression. In this nomogram, the significant variables including the TP53 mutation, age, stage, and TNM status were used to assign points (Figure 7D). The C-index of this nomogram was 0.772, and the calibration plots suggested that there was good consistency between the nomogram and observed OS probabilities in BC (Figure 7E).

**TABLE 5:** Association with overall survival and clinical pathologic characteristics using univariate and multivariate Cox regression.

	Univariate Cox analysis				Multivariate Cox analysis			
	HR	HR.95L	HR.95H	*p* value	HR	HR.95L	HR.95H	*p* value
Age (≥60 vs <60)	1.87	1.33	2.64	0.000324	1.94	1.37	2.76	0.000205
Stage (III + IV + X vs I + II)	2.50	1.77	3.53	1.91E-07	2.46	1.26	4.80	0.008534
T stage (T3 and T4 and TX vs T1andT2)	1.68	1.13	2.49	0.010329	0.87	0.51	1.50	0.627367
M stage (M1 and MX vs M0)	2.30	1.49	3.57	0.000177	1.67	1.05	2.66	0.031934
N stage (N2 and N3 and NX vs N0 and N1)	2.25	1.54	3.30	3.01E-05	1.06	0.57	1.96	0.855684
TP53-mutant (mutant vs. wt)	1.46	1.04	2.06	0.029765	1.76	1.24	2.50	0.001575

## Discussion

TP53 mutations impair its capacity to bind the specific genome sequence that regulates the signaling pathway mediated by TP53 and lead to tumorigenesis and tumor progression in the context of other mutations present in the genome (Baugh et al., 2018). Prior studies revealed a role for TP53 in response to different treatments as complex as its different biological activities (Shahbandi et al., 2020). TP53 mutations contribute to the cancerous phenotype depending on the BC subtype (Silwal-Pandit et al., 2017; Schon and Tischkowitz, 2018). The patients with TP53 mutant tumors had worse survival than patients with TP53 wild-type tumors (Shahbandi et al., 2020). In luminal tumors, inactivation of TP53 via mutation causes the luminal B phenotype and resistance endocrine therapy, whereas mutant TP53 promotes epithelial-mesenchymal transition and stem cell properties in claudin-low and basal-like tumors (Coradini et al., 2012). Nevertheless, the barriers in understanding the clinical implications of TP53 mutations include an insufficient sample size and lack of long-term follow-up data for BC. Thus, we pooled the “Masked Somatic Mutation” datasets of 943 BC patients to analyze the characteristics and potential clinical significance of TP53 mutations, and the data was downloaded from TCGA GDC database. As a result, the TP53 mutation was prevalent in BC tissues and was an independent prognostic factor for poor prognosis. Moreover, we identified that TP53-mutant BCs presented with higher levels of immunogenicity including the ImmuneScore and StromalScore, and lower levels of TIDE than TP53-wt patients. Furthermore, patients with TP53 mutations tended to have richer immunocytes infiltration and more activated subsets in the TME compared to those in TP53-wt BC patients. These results indicated that ICI treatment is more effective in BC patients with TP53 mutations. Through further analysis, the possible mechanism through which TP53 mutations related to the efficacy of ICIs was determined to be its vital role in the tumor immune microenvironment.

We first explored the role of TP53 mutations in BC by assessing the correlation between the gene mutation and the response to immunotherapy in those patients. The results confirmed that the TP53-mutation group showed higher TMB (*p* = 0.037; Figure 2C) and MSI levels (*p* = 0.077; Figure 2D), which suggested that more neoantigens could be recognized by endogenous immune cells, increasing cytotoxicity. This is also in accordance with the findings of previous research. Li et al. found that TP53-mutated cancers were more likely to have a higher level of tumor aneuploidy and TMB than TP53-wt cancers (Li et al., 2020). Moreover, based on the results of retrospective studies, TP53 mutation was found to be a potential biomarker for prognosis and efficacy prediction for BC (Duffy et al., 2018). Further, we also evaluated the TIDE, which is one of the important aspects of the tumor immune escape mechanism (Jiang et al., 2018). Surprisingly, TIDE was also significantly upregulated in the TP53-mutation group (*p* < 0.001; Figure 2E) compared with that in the WT group. This result suggests that the tumor microenvironment of TP53 mutated cancer cells might display an immune escape phenotype in BC. This might be because TP53 is the activator of apoptosis in response to DNA damage that functions by controlling tumor inflammation and immune response, and TP53 mutations could be used to reorganize the tumor immune composition (Blagih et al., 2020).

We further explored the correlation between TP53 status and the proportion of 22 immune cell subtypes in BC. By using the CIBERSORT analysis package, we found that in the TP53-mutant group the proportion of Tregs, T helper cells, and M0 type macrophages was significantly upregulated, whereas the resting CD4^+^ T cell and M2 type macrophages were downregulated. In the TME, TP53 regulates the balance between antigen-presenting cells and myeloid suppressor cells (such as Tregs), and the former could shape the anti-tumor immunity mediated by T cells. In addition, prior studies indicated that the TP53 mutation in tumors could modulate immune recognition by decreasing MHC-I presentation and increasing Treg recruitment (Bezzi et al., 2018). Meanwhile, TP53 mutations can also regulate CD4^+^ T cells recruitment and their immune activity, thus leading to tumor cells escape from immune surveillance and promoting the tumor progression (Wellenstein et al., 2019). We also compared the BC immunogenicity differences between the TP53-mutant and TP53-wt groups. The results demonstrated that in the mutation group, the levels of the ImmuneScore (*p* < 0.001) and StromalScore (*p* = 0.003) were both significantly increased (Figure 6A). This result suggested that the TP53 mutation participated in modulating not only for the immune component, but also the stromal component of TME. Above all, TP53 plays a complex role in TME alterations by promoting the infiltration of diverse immunocytes, thus regulating the progression and prognosis of BC.

To explore the underlying mechanism, we compared differential expression in immune-related hub-genes between TP53-mutant and TP53-wt groups. The results demonstrated that the chemokines CXCL1, CXCL10, and CCL20 were significantly upregulated in the TP53-mutant group. Interestingly, we validated that the expression levels of CXCL1, CXCL10, and CCL20 increased in the TP53-mutant group (*p* < 0.05; Figure E). TP53 mutation modulated the production of cytokines and chemokines in cancer cells, which affect the proportion of immunocytes infiltrating the TME, including neutrophils, Tregs, and macrophages (Bezzi et al., 2018; Wellenstein et al., 2019). Previous findings demonstrated that tumor derived CXCL1 was expressed in stromal cells and epithelial cells, and promoted the cancer growth and its expression level related to the tumor grade (Addadi et al., 2010). Importantly, TP53 in CAFs relieves the repressive effect of chemokine CXCL1, thereby upregulating the migration and angiogenesis of tumor cells (Schauer et al., 2013). Further, macrophages were co-regulated based on TP53 and NF-κB signaling pathways, and TP53 was found to stimulate the secretion of CCL20 and CXCL1, which might facilitate tumor progression (Lowe et al., 2014). However, the TP53 mutation in macrophages either promotes the expression of the proinflammatory cytokines CXCL1 and CCL3, or eliminates the cells by initiating the apoptosis (Lowe et al., 2014). These changes might accelerate the malignant progression of cancer. In addition, the enrichment analysis results indicated that IL-17 signaling pathway was significantly altered in the TP53-mutant group (Table 3), which suggested that TP53 mutation might be involved in reorganizing the TME. Previous studies demonstrated that in BC IL-1β elicits IL-17 expression from γδT cells, and resulted in the polarization of neutrophils, yet the neutralization of IL-17 suppresses the T-cell-suppressive phenotype of neutrophils (Coffelt et al., 2015; Wu et al., 2020). Thus, IL-17 produced by neutrophils and γδT cells acts together to promote the metastasis of BC (Coffelt et al., 2015). These results illustrated that TP53-mutant BC cells were likely to promote the Treg infiltration into TME and secret more chemokines including CXCL1, CXCL10, and CCL20, contributing to several aspects of BC progression.

## Conclusions

In summary, our findings indicate that the TP53 mutation is prevalent in BC and correlates with unfavorable prognosis. Meanwhile, TP53 mutation status is associated with different proportions of immunocytes infiltration, such as Tregs, CD8^+^ T cells, and macrophages. Therefore, TP53 mutations have an essential influence on tumor immune microenvironment and provide a reference to further explore the effective immunotherapy for TP53-mutant BC.

## Data Availability

The datasets presented in this study can be found in online repositories. The names of the repository/repositories and accession number(s) can be found in the article/Supplementary Material.

## References

[B1] Addadi Y. Moskovits N. Granot D. Lozano G. Carmi Y. Apte R. N. (2010). p53 Status in Stromal Fibroblasts Modulates Tumor Growth in an SDF1-dependent Manner. Cancer Res. 70 (23), 9650–9658. 10.1158/0008-5472.CAN-10-1146 20952507 PMC2999653

[B2] Alexandrov L. B. Kim J. Kim J. Haradhvala N. J. Huang M. N. Tian Ng A. W. (2020). The Repertoire of Mutational Signatures in Human Cancer. Nature 578 (7793), 94–101. 10.1038/s41586-020-1943-3 32025018 PMC7054213

[B3] Baugh E. H. Ke H. Levine A. J. Bonneau R. A. Chan C. S. (2018). Why Are There Hotspot Mutations in the TP53 Gene in Human Cancers? Cell Death Differ 25 (1), 154–160. 10.1038/cdd.2017.180 29099487 PMC5729536

[B4] Bezzi M. Seitzer N. Ishikawa T. Reschke M. Chen M. Wang G. (2018). Diverse Genetic-Driven Immune Landscapes Dictate Tumor Progression through Distinct Mechanisms. Nat. Med. 24 (2), 165–175. 10.1038/nm.4463 29309058

[B5] Blagih J. Buck M. D. Vousden K. H. (2020). p53, Cancer and the Immune Response. J. Cel Sci. 133 (5), jcs237453. 10.1242/jcs.237453 32144194

[B6] Britt K. L. Cuzick J. Phillips K.-A. (2020). Key Steps for Effective Breast Cancer Prevention. Nat. Rev. Cancer 20 (8), 417–436. 10.1038/s41568-020-0266-x 32528185

[B7] Coffelt S. B. Kersten K. Doornebal C. W. Weiden J. Vrijland K. Hau C.-S. (2015). IL-17-producing γδ T Cells and Neutrophils Conspire to Promote Breast Cancer Metastasis. Nature 522 (7556), 345–348. 10.1038/nature14282 25822788 PMC4475637

[B8] Colaprico A. Silva T. C. Olsen C. Garofano L. Cava C. Garolini D. (2016). TCGAbiolinks: an R/Bioconductor Package for Integrative Analysis of TCGA Data. Nucleic Acids Res. 44 (8), e71. 10.1093/nar/gkv1507 26704973 PMC4856967

[B9] Coradini D. Fornili M. Ambrogi F. Boracchi P. Biganzoli E. (20122012). TP53Mutation, Epithelial-Mesenchymal Transition, and Stemlike Features in Breast Cancer Subtypes. J. Biomed. Biotechnol. 2012, 1–13. 10.1155/2012/254085 PMC3414214 22899882

[B10] Coughlin S. S. (2019). Epidemiology of Breast Cancer in Women. Adv. Exp. Med. Biol. 1152, 9–29. 10.1007/978-3-030-20301-6_2 31456177

[B11] DeBerardinis R. J. (2020). Tumor Microenvironment, Metabolism, and Immunotherapy. N. Engl. J. Med. 382 (9), 869–871. 10.1056/NEJMcibr1914890 32101671

[B12] Duffy M. J. Synnott N. C. Crown J. (2018). Mutant P53 in Breast Cancer: Potential as a Therapeutic Target and Biomarker. Breast Cancer Res. Treat. 170 (2), 213–219. 10.1007/s10549-018-4753-7 29564741

[B13] Force J. Leal J. H. S. McArthur H. L. (2019). Checkpoint Blockade Strategies in the Treatment of Breast Cancer: where We Are and where We Are Heading. Curr. Treat. Options. Oncol. 20 (4), 35. 10.1007/s11864-019-0634-5 30923913

[B14] Hancock B. A. Chen Y.-H. Solzak J. P. Ahmad M. N. Wedge D. C. Brinza D. (2019). Profiling Molecular Regulators of Recurrence in Chemorefractory Triple-Negative Breast Cancers. Breast Cancer Res. 21 (1), 87. 10.1186/s13058-019-1171-7 31383035 PMC6683504

[B15] Hinshaw D. C. Shevde L. A. (2019). The Tumor Microenvironment Innately Modulates Cancer Progression. Cancer Res. 79 (18), 4557–4566. 10.1158/0008-5472.CAN-18-3962 31350295 PMC6744958

[B16] Hsu S.-D. Lin F.-M. Wu W.-Y. Liang C. Huang W.-C. Chan W.-L. (2011). miRTarBase: a Database Curates Experimentally Validated microRNA-Target Interactions. Nucleic Acids Res. 39 (Database issue), D163–D169. 10.1093/nar/gkq1107 21071411 PMC3013699

[B17] Jiang P. Gu S. Pan D. Fu J. Sahu A. Hu X. (2018). Signatures of T Cell Dysfunction and Exclusion Predict Cancer Immunotherapy Response. Nat. Med. 24 (10), 1550–1558. 10.1038/s41591-018-0136-1 30127393 PMC6487502

[B18] Le Blanc J. M. Heller D. R. Friedrich A. Lannin D. R. Park T. S. (2020). Association of Medicaid Expansion under the Affordable Care Act with Breast Cancer Stage at Diagnosis. JAMA Surg. 155 (8), 752–758. 10.1001/jamasurg.2020.1495 32609338 PMC7330827

[B19] Li L. Li M. Wang X. (2020). Cancer Type-dependent Correlations between TP53 Mutations and Antitumor Immunity. DNA Repair 88, 102785. 10.1016/j.dnarep.2020.102785 32007736

[B20] Li W. Wang H. Ma Z. Zhang J. Ou-Yang W. Qi Y. (2019). Multi-omics Analysis of Microenvironment Characteristics and Immune Escape Mechanisms of Hepatocellular Carcinoma. Front. Oncol. 9, 1019. 10.3389/fonc.2019.01019 31681571 PMC6803502

[B21] Love M. I. Huber W. Anders S. (2014). Moderated Estimation of Fold Change and Dispersion for RNA-Seq Data with DESeq2. Genome Biol. 15 (12), 550. 10.1186/s13059-014-0550-8 25516281 PMC4302049

[B22] Lowe J. M. Menendez D. Bushel P. R. Shatz M. Kirk E. L. Troester M. A. (2014). p53 and NF-Κb Coregulate Proinflammatory Gene Responses in Human Macrophages. Cancer Res. 74 (8), 2182–2192. 10.1158/0008-5472.CAN-13-1070 24737129 PMC3994464

[B23] Maughan K. L. Lutterbie M. A. Ham P. S. (2010). Treatment of Breast Cancer. Am. Fam. Physician 81 (11), 1339–1346. 20521754

[B24] Mayakonda A. Lin D.-C. Assenov Y. Plass C. Koeffler H. P. (2018). Maftools: Efficient and Comprehensive Analysis of Somatic Variants in Cancer. Genome Res. 28 (11), 1747–1756. 10.1101/gr.239244.118 30341162 PMC6211645

[B25] Newman A. M. Liu C. L. Green M. R. Gentles A. J. Feng W. Xu Y. (2015). Robust Enumeration of Cell Subsets from Tissue Expression Profiles. Nat. Methods 12 (5), 453–457. 10.1038/nmeth.3337 25822800 PMC4739640

[B26] Pitolli C. Wang Y. Mancini M. Shi Y. Melino G. Amelio I. (2019). Do mutations Turn P53 into an Oncogene? Ijms 20 (24), 6241. 10.3390/ijms20246241 PMC6940991 31835684

[B27] Reich M. Liefeld T. Gould J. Lerner J. Tamayo P. Mesirov J. P. (2006). GenePattern 2.0. Nat. Genet. 38 (5), 500–501. 10.1038/ng0506-500 16642009

[B28] Ringnér M. (2008). What Is Principal Component Analysis? Nat. Biotechnol. 26 (3), 303–304. 10.1038/nbt0308-303 18327243

[B29] Robin X. Turck N. Hainard A. Tiberti N. Lisacek F. Sanchez J.-C. (2011). pROC: an Open-Source Package for R and S+ to Analyze and Compare ROC Curves. BMC Bioinformatics 12, 77. 10.1186/1471-2105-12-77 21414208 PMC3068975

[B30] Santa-Maria C. A. Nanda R. (2018). Immune Checkpoint Inhibitor Therapy in Breast Cancer. J. Natl. Compr. Canc. Netw. 16 (10), 1259–1268. 10.6004/jnccn.2018.7046 30323094

[B31] Schauer I. G. Zhang J. Xing Z. Guo X. Mercado-Uribe I. Sood A. K. (2013). Interleukin-1β Promotes Ovarian Tumorigenesis through a p53/NF-Κb-Mediated Inflammatory Response in Stromal Fibroblasts. Neoplasia 15 (4), 409–IN18. 10.1593/neo.121228 23555186 PMC3612913

[B32] Schon K. Tischkowitz M. (2018). Clinical Implications of Germline Mutations in Breast Cancer: TP53. Breast Cancer Res. Treat. 167 (2), 417–423. 10.1007/s10549-017-4531-y 29039119 PMC5790840

[B33] Shahbandi A. Nguyen H. D. Jackson J. G. (2020). TP53 Mutations and Outcomes in Breast Cancer: reading beyond the Headlines. Trends Cancer 6 (2), 98–110. 10.1016/j.trecan.2020.01.007 32061310 PMC7931175

[B34] Shannon P. Markiel A. Ozier O. Baliga N. S. Wang J. T. Ramage D. (2003). Cytoscape: a Software Environment for Integrated Models of Biomolecular Interaction Networks. Genome Res. 13 (11), 2498–2504. 10.1101/gr.1239303 14597658 PMC403769

[B35] Silwal-Pandit L. Langerød A. Børresen-Dale A.-L. (2017). TP53Mutations in Breast and Ovarian Cancer. Cold Spring Harb. Perspect. Med. 7 (1), a026252. 10.1101/cshperspect.a026252 27815305 PMC5204332

[B36] Speir M. L. Zweig A. S. Rosenbloom K. R. Raney B. J. Paten B. Nejad P. (2016). The UCSC Genome Browser Database: 2016 Update. Nucleic Acids Res. 44 (D1), D717–D725. 10.1093/nar/gkv1275 26590259 PMC4702902

[B37] Subramanian A. Tamayo P. Mootha V. K. Mukherjee S. Ebert B. L. Gillette M. A. (2005). Gene Set Enrichment Analysis: a Knowledge-Based Approach for Interpreting Genome-wide Expression Profiles. Proc. Natl. Acad. Sci. 102 (43), 15545–15550. 10.1073/pnas.0506580102 16199517 PMC1239896

[B38] Szklarczyk D. Gable A. L. Lyon D. Junge A. Wyder S. Huerta-Cepas J. (2019). STRING V11: Protein-Protein Association Networks with Increased Coverage, Supporting Functional Discovery in Genome-wide Experimental Datasets. Nucleic Acids Res. 47 (D1), D607–D613. 10.1093/nar/gky1131 30476243 PMC6323986

[B39] Vilar E. Gruber S. B. (2010). Microsatellite Instability in Colorectal Cancer-The Stable Evidence. Nat. Rev. Clin. Oncol. 7 (3), 153–162. 10.1038/nrclinonc.2009.237 20142816 PMC3427139

[B40] Wang X. Xing L. Yang R. Chen H. Wang M. Jiang R. (2021). The circACTN4 Interacts with FUBP1 to Promote Tumorigenesis and Progression of Breast Cancer by Regulating the Expression of Proto-Oncogene MYC. Mol. Cancer 20 (1), 91. 10.1186/s12943-021-01383-x 34116677 PMC8194204

[B41] Wellenstein M. D. Coffelt S. B. Duits D. E. M. van Miltenburg M. H. Slagter M. de Rink I. (2019). Loss of P53 Triggers WNT-dependent Systemic Inflammation to Drive Breast Cancer Metastasis. Nature 572 (7770), 538–542. 10.1038/s41586-019-1450-6 31367040 PMC6707815

[B42] Wu L. Awaji M. Saxena S. Varney M. L. Sharma B. Singh R. K. (2020). IL-17-CXC Chemokine Receptor 2 axis Facilitates Breast Cancer Progression by Up-Regulating Neutrophil Recruitment. Am. J. Pathol. 190 (1), 222–233. 10.1016/j.ajpath.2019.09.016 31654638 PMC6943375

[B43] Yang W. Soares J. Greninger P. Edelman E. J. Lightfoot H. Forbes S. (2013). Genomics of Drug Sensitivity in Cancer (GDSC): a Resource for Therapeutic Biomarker Discovery in Cancer Cells. Nucleic Acids Res. 41 (Database issue), D955–D961. 10.1093/nar/gks11111 23180760 PMC3531057

[B44] Yarchoan M. Hopkins A. Jaffee E. M. (2017). Tumor Mutational burden and Response Rate to PD-1 Inhibition. N. Engl. J. Med. 377 (25), 2500–2501. 10.1056/NEJMc1713444 29262275 PMC6549688

[B45] Yoshihara K. Shahmoradgoli M. Martínez E. Vegesna R. Kim H. Torres-Garcia W. (2013). Inferring Tumour Purity and Stromal and Immune Cell Admixture from Expression Data. Nat. Commun. 4, 2612. 10.1038/ncomms3612 24113773 PMC3826632

[B46] Yu G. Wang L.-G. Han Y. He Q.-Y. (2012). clusterProfiler: an R Package for Comparing Biological Themes Among Gene Clusters. OMICS: A J. Integr. Biol. 16 (5), 284–287. 10.1089/omi.2011.0118 PMC3339379 22455463

[B47] Zhang J. Bajari R. Andric D. Gerthoffert F. Lepsa A. Nahal-Bose H. (2019). The International Cancer Genome Consortium Data portal. Nat. Biotechnol. 37 (4), 367–369. 10.1038/s41587-019-0055-9 30877282

[B48] Zhang Z. Hernandez K. Savage J. Li S. Miller D. Agrawal S. (2021). Uniform Genomic Data Analysis in the NCI Genomic Data Commons. Nat. Commun. 12 (1), 1226. 10.1038/s41467-021-21254-9 33619257 PMC7900240

