# Supplementary figures and images for: TP53 Mutation Infers a Poor Prognosis and Is Correlated to Immunocytes Infiltration in Breast Cancer
Source: Front Cell Dev Biol. 2021 Nov 30;9:759154. doi: 10.3389/fcell.2021.759154 (PMC8669954; doi:10.3389/fcell.2021.759154)

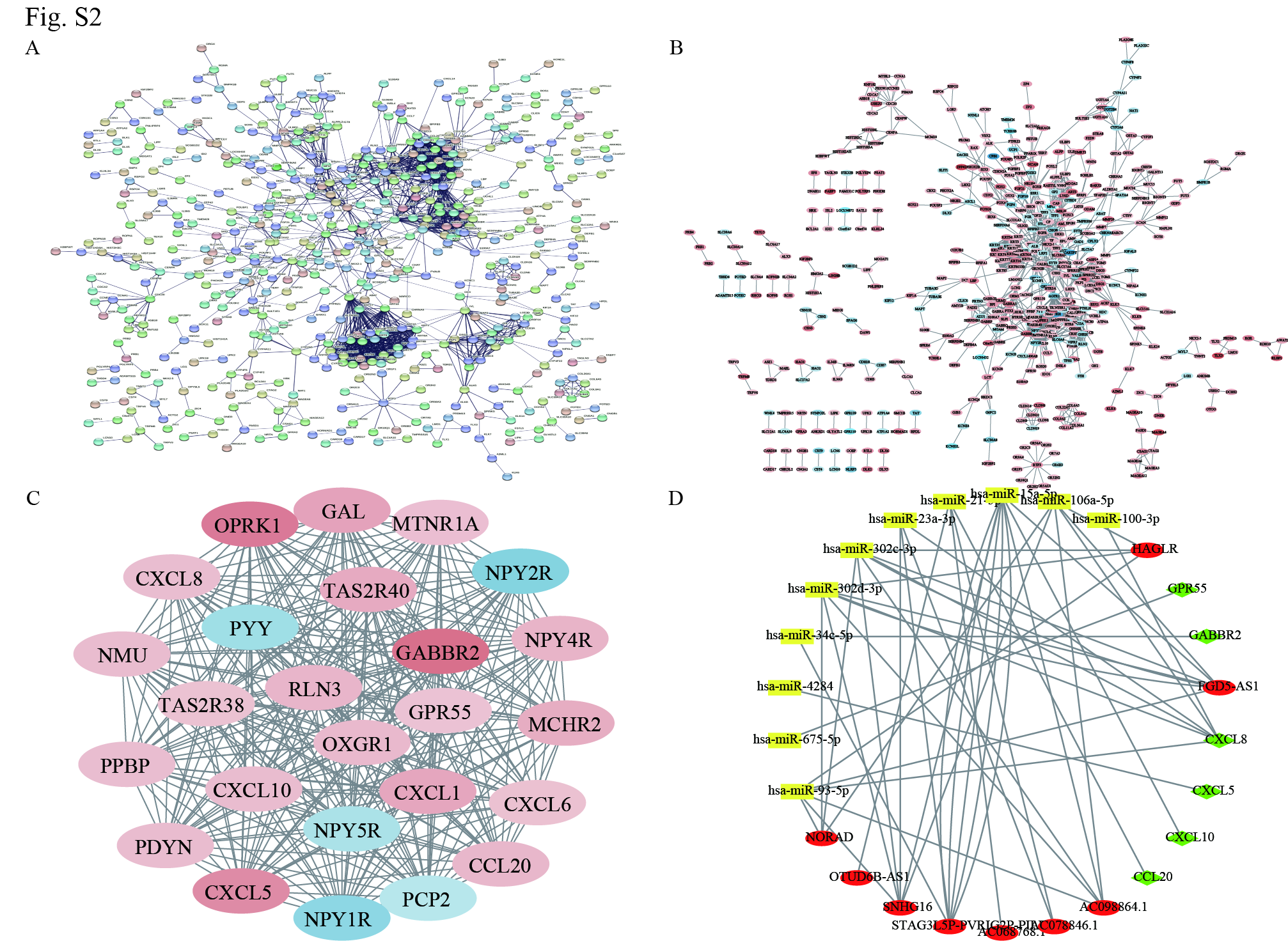

Supplement: Supplementary file 2 [file Image2.TIF]

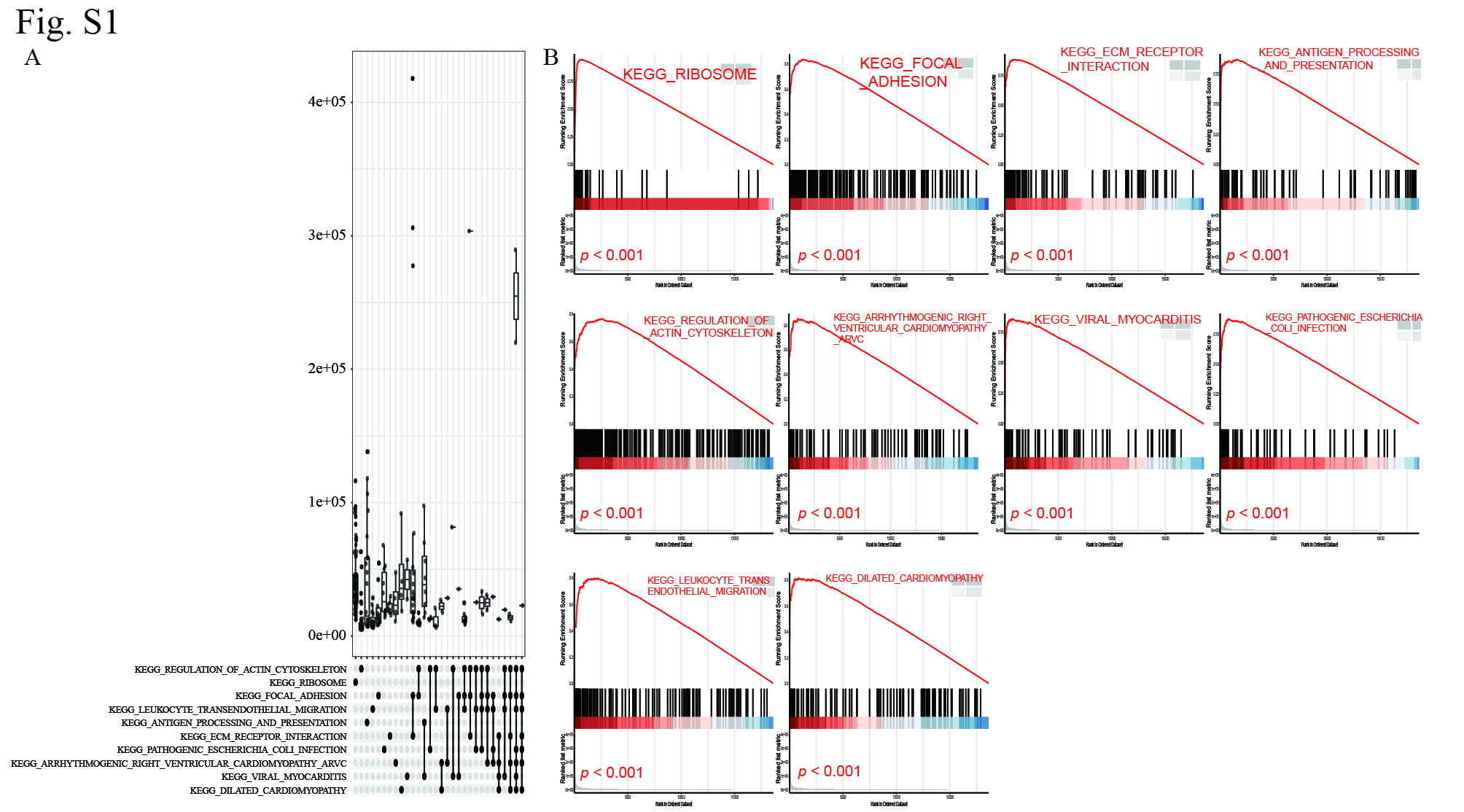

Supplement: Supplementary file 3 [file Image1.TIF]
